# Increased free water in the nucleus basalis of Meynert is associated with worse olfaction in idiopathic Parkinson’s disease

**DOI:** 10.3389/fnagi.2025.1689150

**Published:** 2025-11-11

**Authors:** Kai Wei, Shuyue Wang, Cheng Zhou, Peiyu Huang, Chao Wang

**Affiliations:** Department of Radiology, The Second Affiliated Hospital, Zhejiang University School of Medicine, Hangzhou, Zhejiang, China

**Keywords:** Parkinson’s disease, cholinergic basal forebrain, nucleus basalis of Meynert, free water, olfaction

## Abstract

**Background:**

The association of cholinergic degeneration and olfactory function in Parkinson’s disease (PD) is unclear. Using free water imaging, this study aims to investigate the association between the nucleus basalis of Meynert (NBM) degeneration and olfactory function in patients with PD.

**Methods:**

A total of 281 idiopathic patients with PD and 98 healthy controls with diffusion tensor MRI data were included from the Parkinson’s Progression Markers Initiative (PPMI) dataset. Olfactory function was assessed by the University of Pennsylvania Smell Identification Test (UPSIT). Free water and volume of the NBM were measured and compared between the two groups. Furthermore, correlation analyses were performed to determine the association between the free water and volume of the NBM and clinical measures in patients with PD. Then, the discriminative power of the free water was evaluated between PD patients with hyposmia and without hyposmia by receiver operating characteristic curve (ROC) analysis.

**Results:**

Nucleus basalis of Meynert free water showed a trend toward increase in PD patients compared with healthy controls (*p* = 0.064), while NBM volume showed no significant difference between patients with PD and healthy controls (*p* = 0.393). Pearson correlation analyses revealed significant correlations between age and free water (*r* = 0.252, *p* < 0.001) and volume (*r* = −0.48, *p* < 0.001) of NBM in patients with PD. Pearson correlation analyses showed NBM free water was negatively correlated with UPSIT scores (*p* < 0.001), while NBM volume was positively correlated with UPSIT scores (*p* = 0.002) in patients with PD. Partial correlation analyses were further performed adjusting for age, the results showed NBM free water remained significantly negatively correlated with UPSIT score (*p* = 0.013), while NBM volume was not significantly correlated with UPSIT score in patients with PD (*p* = 0.461). In addition, ROC analysis showed that NBM free water identified PD patients with hyposmia at high sensitivity (81.6%).

**Conclusion:**

Our study demonstrated that the free water of the NBM was associated with worse olfaction in idiopathic patients with PD. Our study suggests that free water in the NBM has the potential to provide early biomarkers of olfaction dysfunction in idiopathic patients with PD.

## Introduction

Parkinson’s disease (PD) ranks as the second most common neurodegenerative condition, impacting approximately 2%–3% of the population aged 65 and older (Poewe et al., 2017). The primary neuropathological features of PD include degeneration of substantia nigra neurons, leading to reduced striatal dopamine levels, and intracellular accumulation of α-synuclein protein, both of which contribute to nigrostriatal pathway dysfunction (Dickson et al., 2009). Olfactory dysfunction in PD is characterized by impairments in identifying odors, discriminating between different odors, detecting odor thresholds, and remembering familiar odors (Mesholam et al., 1998; Haehner et al., 2009). In both patients with PD and rat models, impaired olfaction has been identified to be associated with a reduction in cholinergic innervation. In patients with PD, a study has revealed a significant correlation between impaired olfaction and cortical cholinergic denervation evaluated by the short latency afferent inhibition response (Oh et al., 2017). In rat models, both selective lesioning of cholinergic basal forebrain neurons with 192 IgG-saporin (Linster et al., 2001) and infusing a nicotinic antagonist (mecamylamine hydrochloride) into the olfactory bulbs (Mandairon et al., 2006) could injure olfactory discrimination. In contrast, the acetylcholinesterase inhibitor (neostigmine) could enhance olfactory discrimination in rat models (Mandairon et al., 2006). These findings provide evidence supporting the notion that impaired olfaction serves as a clinical indicator of cholinergic denervation. These investigations provided evidence to suggest that the presence of olfactory dysfunction may serve as a clinical indicator of cholinergic denervation. The nucleus basalis of Meynert (NBM), within the cholinergic basal forebrain, provides the primary cholinergic input to the cortex. Thus, reduced cholinergic innervation from the basal forebrain, particularly the NBM, may contribute to hyposmia in PD.

Free water imaging employs a bi-tensor model to explicitly estimate the fractional volume of freely diffused water molecules within the voxel based on diffusion tensor imaging (Pasternak et al., 2009). Increased free water is associated with heightened neuroinflammation, damage to axons/myelin, and degenerative processes linked to atrophy-based neurodegeneration (Pasternak et al., 2009; Wang et al., 2011; Febo et al., 2020). Free water imaging of the substantia nigra (SN) is emerging as a promising biomarker for distinguishing patients with PD from healthy controls (Ofori et al., 2017), as well as for monitoring disease progression of PD (Ofori et al., 2017; Zhou et al., 2023). However, it remains unclear about the association of the NBM degeneration, especially the free water changes and olfactory function in patients with PD. Therefore, the primary aim of this study is to investigate the association between the NBM degeneration (i.e., free water and volume changes) and olfactory function assessed by the University of Pennsylvania Smell Identification Test (UPSIT) in patients with PD. As the decrease of dopamine transporter (DAT) binding capacity and dopamine concentration are the neuropathological hallmarks of PD due to degeneration of dopaminergic neurons (Uhl, 2003), the secondary aim of this study is to investigate the association between the NBM degeneration (i.e., free water and volume changes) and DAT binding.

## Materials and methods

### Participants

**FIGURE 1:**
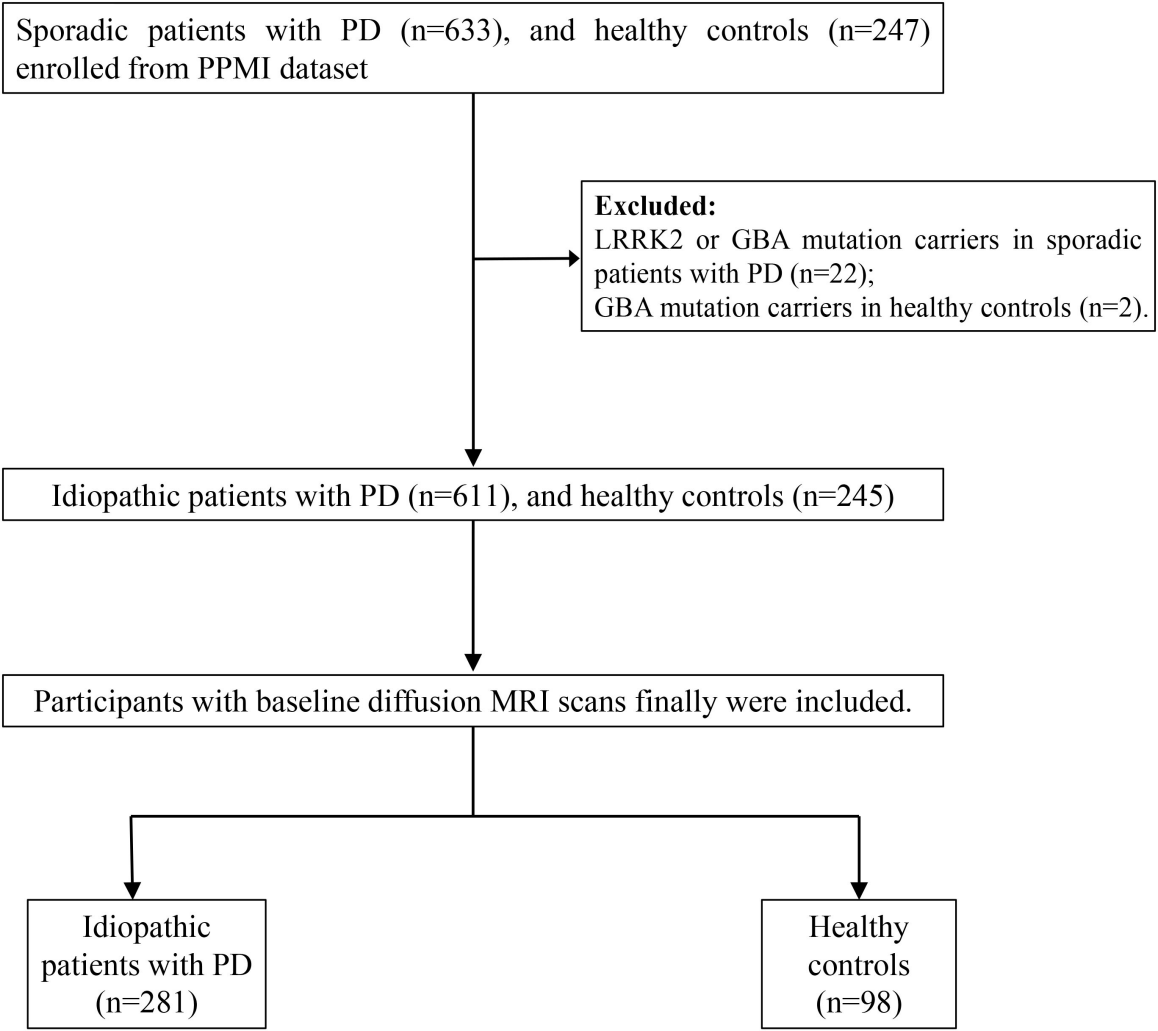
The flowchart shows participant enrollment. LRRK2, leucine-rich repeat kinase 2 mutations; GBA, glucocerebrosidase.

All participants were enrolled from the Parkinson’s Progression Markers Initiative (PPMI) database^1^. PPMI is an ongoing, multi-site, global observational study designed to discover biomarkers related to Parkinson’s disease (PD) progression, including those derived from blood, genetics, cerebrospinal fluid, and neuroimaging. Detailed study protocols and guidelines are accessible online. In the PPMI cohort, newly diagnosed, untreated PD patients and healthy individuals were recruited according to previously established inclusion and exclusion criteria (Marek et al., 2018). At each participating PPMI center, the study was reviewed and approved by the Institutional Review Board, and all participants provided written consent before enrollment. In this study, we included newly diagnosed, untreated idiopathic PD patients with baseline diffusion MRI, and excluded the LRRK2 or GBA mutation PD patients. A diagram illustrating the participant selection process is presented in Figure 1.

### Clinical evaluations

All participants underwent the PPMI standard test battery of assessments. Demographic data such as gender, age, and educational background were gathered. Olfactory function was assessed using the UPSIT, which has 40 odors identified from scratch-and-sniff panels with a forced choice from four options per odor. The total score ranges from 0 (no odor correctly identified) to 40 (all odors correctly identified). Among patients with PD, patients scoring below 24 in UPSIT were classified as patients with hyposmia, while patients scoring at or above 24 in UPSIT were classified as patients without hyposmia (Conti et al., 2013; Devanand et al., 2015; Kanavou et al., 2021). The other clinical assessments used in the analysis included the Hoehn and Yahr (H-Y) stages, Movement Disorders Society Unified Parkinson’s Disease Rating Scale (MDS-UPDRS), Montreal Cognitive Assessment (MOCA), Geriatric Depression Scale (GDS), Epworth sleepiness scale (ESS), Rapid Eye Movement Behavior Disorder Screening Questionnaire (RBDSQ), and Scales for Outcomes in Parkinson’s Disease-Autonomic Function (SCOPA-AUT). Furthermore, all participants were required to undergo a DAT scan to evaluate striatal binding ratios (SBR) in the caudate nucleus and putamen, with analyses conducted in accordance with the PPMI imaging technical operations guidelines.

### Magnetic resonance imaging acquisition

Magnetic resonance imaging data were acquired using 3.0 T MRI scanners from several manufacturers, including TrioTim/Prisma/Verio/Biograph/Skyra models from Siemens (Germany), Achieva dStream and Ingenia scanners from Philips Medical Systems (Netherlands), and SIGNA Architect/DISCOVERY MR750 scanners from General Electric Company (GE) Medical Systems (USA). Before scanning commenced, each participating site was provided with an electronic protocol tailored to the specific software version, which was then uploaded into the respective MRI scanner. The DTI sequence parameters were as follows: 64 diffusion gradient directions; one b0 image; *b*-value = 1000 s/mm^2^; voxel size = 1.983 mm × 1.983 mm × 2.0 mm; matrix = 116 × 116 × 72; flip angle = 90°. Structural 3D-T1-weighted images were obtained using a magnetization-prepared rapid gradient-echo (MPRAGE) sequence with the following parameters: isotropic voxel dimensions of 1 mm × 1 mm × 1 mm, a flip angle of 9°, echo time (TE) ranging from 2.93 to 2.98 ms, repetition time (TR) of 2300 ms, and inversion time (TI) of 900 ms.

### Magnetic resonance image analysis

**FIGURE 2:**
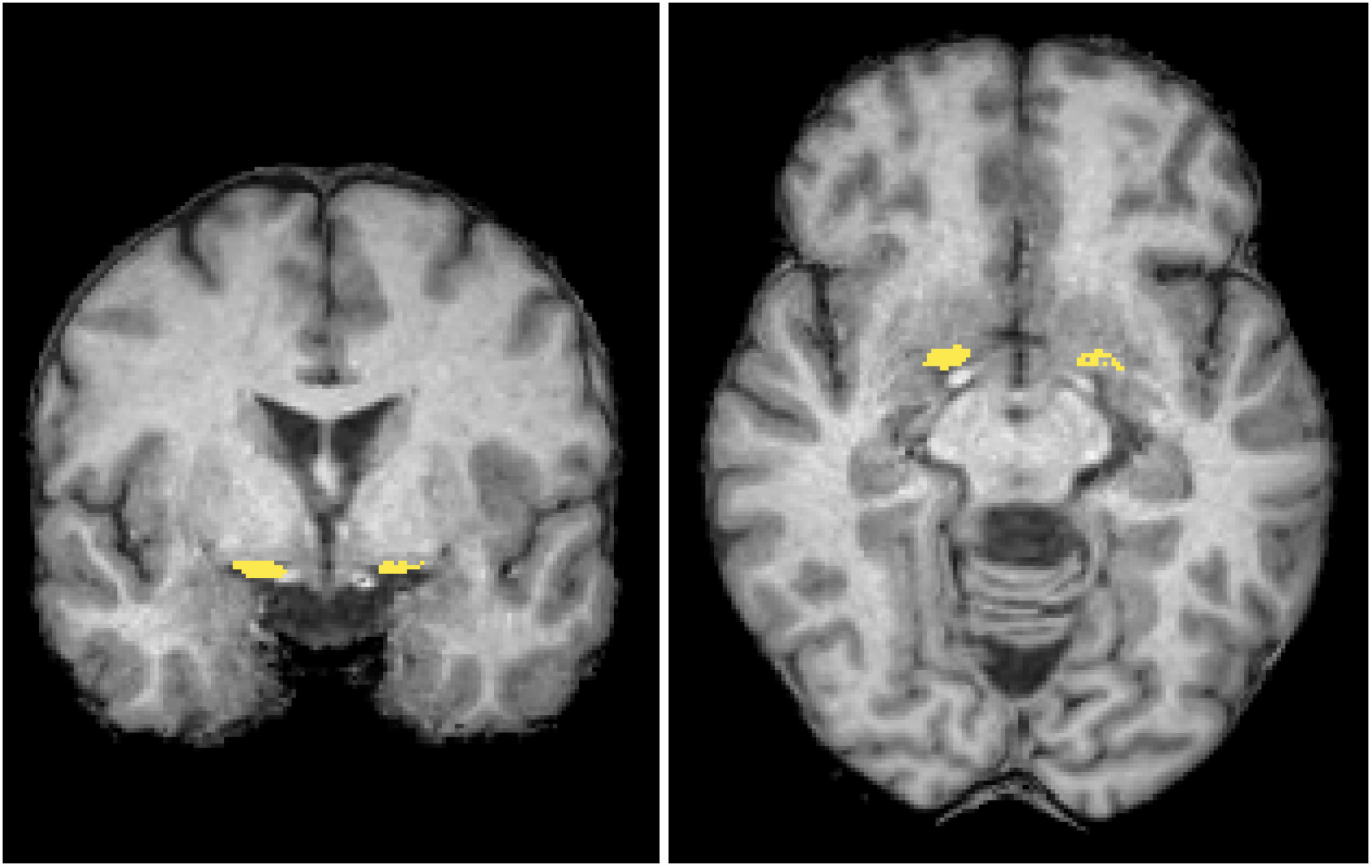
Regions of interest. The mask of the nucleus basalis of Meynert (NBM) (yellow) is overlaid in coronal and axial views on a standard brain T1-weighted images in the MNI space. MNI, Montreal Neurological Institute.

Morphometry analysis was conducted using SPM12^2^ and CAT12^3^. The 3D T1-weighted images were segmented into distinct tissue types and spatially normalized to the standard template through Diffeomorphic Anatomical Registration via Exponentiated Lie Algebra (DARTEL). The regions of interest (ROIs) of the NBM (Figure 2) were delineated based on the Julich-Brain Cytoarchitectonic Atlas (Amunts et al., 2020), which was developed using high-resolution imaging of histological sections from 23 postmortem human brains. To facilitate diffusion analysis, ROIs in the standard space were mapped back to each individual’s anatomical space by applying the inverse deformation field obtained from DARTEL. During spatial normalization, segmented maps were modulated by the Jacobian determinants of the deformation field to preserve local volumetric information. We then computed NBM volume by summing, within the ROI, the voxelwise product of the modulated segmentation and the NBM probability map.

The DTI image preprocessing was carried out with MRtrix3^4^ and the FMRIB Software Library (FSL, version 6.0)^5^, involving steps such as noise reduction, Gibbs artifact suppression, motion correction, and eddy current adjustment. Subsequently, the free water model was computed using a script provided by the MarkVCID project^6^. The model estimated two distinct compartments: one representing the free water compartment, which captures isotropic diffusion primarily associated with water molecules in the extracellular environment, and the other representing the tissue compartment, which characterizes the underlying tissue microstructure after excluding the signal arising from free water. The fractional volume of the free water compartment—referred to as the free water metric—indicates the proportion of free water within each voxel, with values ranging from 0 to 1. It has been demonstrated to be a sensitive measure for detecting microstructure alterations in the NBM (Wang et al., 2024; Zhang et al., 2024). For each participant, the b0 images were spatially aligned to the corresponding T1-weighted images using the epi_reg tool in FSL. This tool is specifically developed for registering functional or diffusion data to structural images, employing a boundary-based cost function to enhance registration accuracy. The obtained transformation matrix was subsequently inverted and utilized to map the ROIs from the structural space into the diffusion image space. To minimize potential contamination from neighboring white matter, further voxel selection criteria were applied to the ROIs, based on methodologies from earlier research (Schulz et al., 2018; Ray et al., 2023). Specifically, fractional anisotropy (FA) images were generated using the dtifit tool in FSL. For the NBM, voxels with FA values exceeding 0.3 were excluded, following the approach outlined in an earlier study (Ray et al., 2023). Afterward, mean free water values of the NBM were computed for each ROI, with values from corresponding bilateral ROIs being averaged. Lastly, to account for variability arising from the use of multiple sites or scanners, the COMBAT method was employed to harmonize the free water measurements (Fortin et al., 2017; Eshaghzadeh Torbati et al., 2021).

### Statistical analysis

For demographic and clinical characteristics and SBR and free water values, independent samples *t*-tests were used to compare continuous variables that did follow a normal distribution, Mann–Whitney U tests were used to compare continuous variables that did not follow a normal distribution. In addition, the χ^2^ test was used for categorical variables. The significance threshold was set at *p* < 0.05.

Pearson correlation analyses were performed to determine whether there was a relationship between NBM free water/volume and clinical characteristics in patients with PD. In addition, partial correlation analyses adjusting for age were performed to determine whether there was a relationship between NBM free water/volume and clinical characteristics in patients with PD. In addition, we employed receiver operating characteristic curve (ROC) analyses to examine whether the free water values of NBM were able to differentiate patients with and without hyposmia. Statistical analyses were done using SPSS (version 26.0, Chicago, IL, USA).

## Results

A total of 281 idiopathic patients with PD and 98 healthy controls were finally included. No differences in demographics were noted between the study groups, including age, gender, and education (Table 1). MDS-UPDRS I, II, III, UPSIT, RBDSQ, and SCOPA-AUT scores differed significantly between groups (*p* < 0.001). Compared to healthy controls, patients with PD showed significantly decreased SBR values in the caudate and putamen (*p* < 0.001).

**TABLE 1:** Demographic and clinical characteristics, and SBR and FW values between patients with Parkinson’s disease and healthy controls.

	Healthy controls (*n* = 98)	Parkinson’s disease (*n* = 281)	*p*-value
Age (years), mean (SD)	61.8 (11.4)	62.9 (9.2)	0.415
Women, *n* (%)	41 (41.8)	94 (33.5)	0.136
Education (years), median (IQR)	16 (13–18)	16 (14–18)	0.411
H-Y stage, median (IQR)	NA	2 (1–2)	NA
Disease duration (months), median (IQR)	NA	5 (3–11)	NA
MDS-UPDRS part I, median (IQR)	2 (0–3)	5 (3–8)	<0.001
MDS-UPDRS part II, median (IQR)	0 (0–0)	5 (2–8)	<0.001
MDS-UPDRS part III, median (IQR)	0 (0–1)	20 (14.5–28.5)	<0.001
UPSIT score, median (IQR)	33 (30–37)	23 (17–29)	<0.001
MOCA score, median (IQR)	28 (27–29)	28 (26–29)	0.028
RBDSQ score, median (IQR)	2 (1–4)	3 (2–5)	<0.001
ESS score, median (IQR)	5 (3–8)	5 (3–7)	0.451
SCOPA-AUT score, median (IQR)	5 (4–8)	8 (5–13)	<0.001
GDS score, median (IQR)	5 (5–6)	5 (5–6)	0.325
SBR-Cau, mean (SD)	3.02 (0.60)	2.00 (0.60)	<0.001
SBR-Puta, mean (SD)	2.19 (0.52)	0.87 (0.29)	<0.001

H-Y, Hoehn and Yahr; MDS-UPDRS, Movement Disorders Society Unified Parkinson’s Disease Rating Scale; MOCA, Montreal Cognitive Assessment; RBDSQ, Rapid Eye Movement Sleep Behavior Disorder Screening Questionnaire; ESS, Epworth sleepiness scale; UPSIT, University of Pennsylvania Smell Identification Test; SCOPA-AUT, Scales for Outcomes in Parkinson’s Disease-Autonomic Function; GDS, Geriatric Depression Scale; SBR, striatal binding ratio; Cau, caudate nucleus; Puta, putamen; NA, not applicable; FW, free water. For variables presented as mean (SD), an independent samples *t*-test was used. For variables presented as numbers (percent), the χ2 test was used. For variables presented as median (IQR), the Mann-Whitney test was used.

**FIGURE 3:**
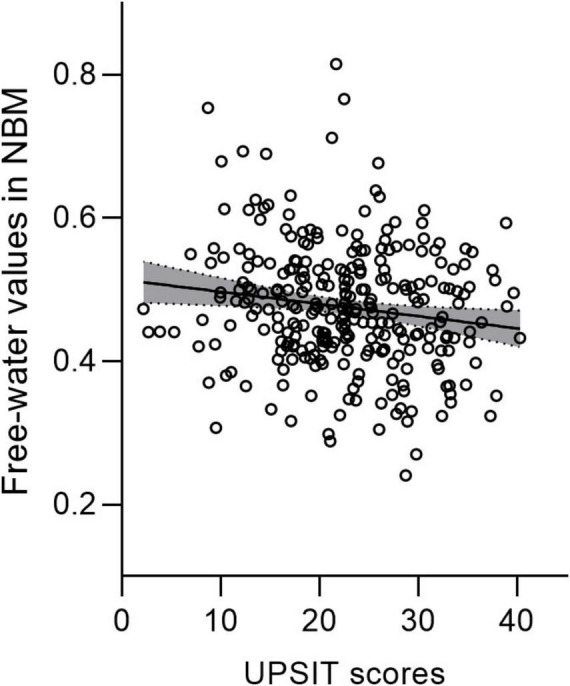
Free water values in the nucleus basalis of Meynert (NBM) are negatively correlated with UPSIT scores in patients with PD. PD, Parkinson’s disease; UPSIT, University of Pennsylvania Smell Identification Test.

**FIGURE 4:**
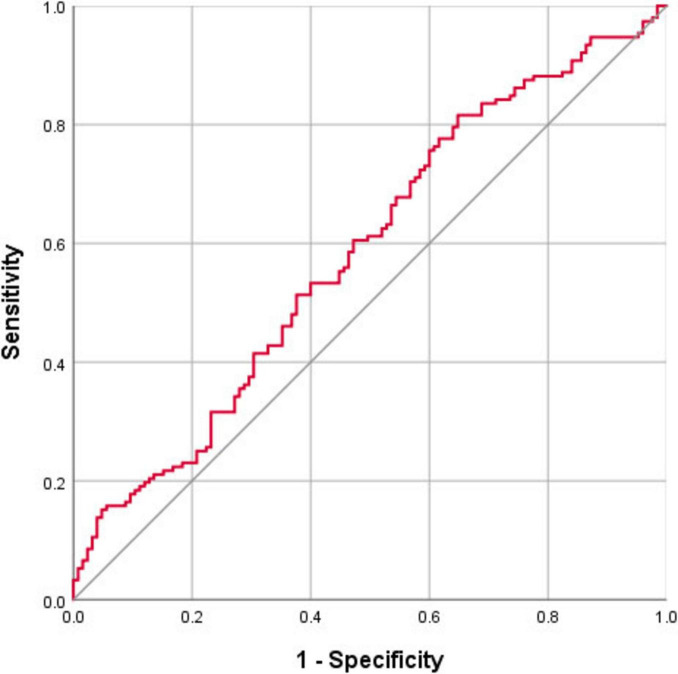
ROC curve showing the classification accuracy in distinguishing patients with and without hyposmia using the free water values of the nucleus basalis of Meynert (NBM). The area under the curve (AUC) is 0.59 (*p* = 0.015, cutoff value = 0.419, sensitivity = 81.6%, specificity = 35.2%).

Nucleus basalis of Meynert free water showed a trend toward increase in PD patients compared with healthy controls (*p* = 0.064), whereas NBM volume did not differ significantly between groups (*p* = 0.393) (Table 2). Pearson correlation analyses revealed significant correlations between age and free water (*r* = 0.252, *p* < 0.001) and volume (*r* = −0.48, *p* < 0.001) of the NBM in patients with PD (Table 3). NBM free water was negatively correlated with UPSIT scores (*p* < 0.001), while NBM volume was positively correlated with UPSIT scores (*p* = 0.002) in patients with PD (Table 3). In addition, partial correlation analyses were further performed adjusting for age, the results showed NBM free water remained significantly negatively correlated with UPSIT score (*p* = 0.013; Figure 3), while NBM volume was not significantly correlated with UPSIT score in patients with PD (*p* = 0.461). In addition, ROC analysis was performed to evaluate the distinguishing power of NBM free water between patients with and without hyposmia. The area under the curve (AUC) was 0.59 (*p* = 0.015, 95% confidence interval [CI] = 0.518–0.653, sensitivity = 81.6%, specificity = 35.2%, cutoff value = 0.419) (Figure 4).

**TABLE 2:** Comparison of free water and volume of the nucleus basalis of Meynert between patients with Parkinson’s disease and healthy controls.

	Healthy controls	Parkinson’s disease	*p*-value
NBM FW, mean (SD, SE)	0.457 (0.086, 0.0086)	0.476 (0.089, 0.0053)	0.064
NBM volume, mean (SD, SE)	232.6 (22.9, 2.3)	234.9 (23.3, 1.4)	0.393

FW, free water values; SD, standard deviation; SE, standard errors.

**TABLE 3:** Pearson correlation analyses between free water and volume of the nucleus basalis of Meynert and clinical characteristics in patients with Parkinson’s disease.

	r (FW)	*p* (FW)	r (volume)	*p* (volume)
Age	0.252	**<0.001**	−0.48	**<0.001**
Education	0.015	0.807	0.106	0.076
Disease duration	0.040	0.502	−0.026	0.659
MDS-UPDRS part I	0.047	0.433	−0.233	**<0.001**
MDS-UPDRS part II	0.076	0.203	−0.177	**0.003**
MDS-UPDRS part III	0.128	0.032	−0.213	**<0.001**
MOCA	−0.065	0.278	0.134	**0.025**
RBDSQ	0.063	0.295	−0.067	0.265
ESS	0.117	0.051	−0.142	**0.017**
UPSIT	−0.214	**<0.001**	0.185	**0.002**
SCOPA-AUT	0.061	0.307	−0.179	**0.003**
GDS	0.051	0.399	−0.026	0.659
SBR-Cau	−0.138	**0.022**	0.145	**0.016**
SBR-Puta	−0.120	**0.045**	0.124	**0.039**

Bold font indicates significant differences. FW, free water values; MDS-UPDRS, Movement Disorders Society Unified Parkinson’s Disease Rating Scale; MOCA, Montreal Cognitive Assessment; RBDSQ, Rapid Eye Movement Sleep Behavior Disorder Screening Questionnaire; ESS, Epworth sleepiness scale; UPSIT, University of Pennsylvania Smell Identification Test; SCOPA-AUT, Scales for Outcomes in Parkinson’s Disease-Autonomic Function; GDS, Geriatric Depression Scale; SBR, striatal binding ratio; Cau, caudate nucleus; Puta, putamen.

Furthermore, compared to healthy controls, patients with PD showed significantly decreased SBR values in the caudate and putamen (*p* < 0.001) (Table 1). NBM free water was negatively correlated with SBR values in the caudate (*p* = 0.022) and putamen (*p* = 0.045), while NBM volume was positively correlated with SBR values in the caudate (*p* = 0.016) and putamen (*p* = 0.039) (Table 3).

## Discussion

This study revealed a trend toward increased NBM free water in PD patients compared with healthy controls, whereas NBM volume did not differ significantly between groups. Pearson correlation analyses revealed significant correlations between age and free water values and volume of NBM in patients with PD. Partial correlation analyses were further performed adjusting for age, the results showed NBM free water remained significantly negatively correlated with UPSIT score, while NBM volume was not significantly correlated with UPSIT score in patients with PD. ROC analysis demonstrated that NBM free water identified PD patients with hyposmia at high sensitivity (81.6%). In addition, compared to healthy controls, patients with PD showed significantly decreased DAT binding in the caudate and putamen. NBM free water was negatively correlated with DAT binding, while NBM volume was positively correlated with DAT binding in the caudate and putamen.

Free water imaging is emerging as an essential tool in the development of biomarkers for neurodegenerative diseases. This imaging parameter is expected to exhibit an increase in cases of neuroinflammation and atrophy-related neurodegeneration (Wang et al., 2011; Febo et al., 2020). During the process of PD pathology, Lewy pathology formation may lead to neurotoxicity, inflammation, oxidative stress, edema, demyelination, and neurodegeneration, which in turn may result in the accumulation of free water molecules in the extracellular space (Pasternak et al., 2009; Ofori et al., 2015; Zhou et al., 2021). Recently, a study showed that free water values increased with age in the white matter (Pieciak et al., 2023). Moreover, a previous PET study found that cholinergic activity decreased with age (Liu et al., 2018). In this study, we further indicated that free water values in the NBM increase with age.

In this study, we found that increased NBM free water was correlated with worse olfaction in patients with PD. Lewy bodies and neuronal loss in the SN are regarded as the gold standard for PD neuropathology, first recognized by Friedrich Lewy in the NBM of postmortem brain tissue in 1913. NBM is recognized as one of the earliest sites of neurodegeneration, which is located in the substantia innominata of the cholinergic basal forebrain (Liu et al., 2015). Degeneration of the NBM, which provides cholinergic innervation to the entire neocortex, is a characteristic of PD and PD dementia (Candy et al., 1983; Perry et al., 1985). A recent study has applied free water imaging and found that free water values in the NBM are associated with current and future changes to cognition in patients with PD (Ray et al., 2023). In both patients with PD and animal models, previous evidence has demonstrated that the presence of olfactory dysfunction can serve as a clinical indicator of cholinergic denervation (Doty et al., 1999; Linster et al., 2001; Oh et al., 2017; Bohnen et al., 2022). The NBM is the primary cholinergic nucleus within the cholinergic basal forebrain. During the disease course of PD, there are Lewy body aggregates and neuronal loss in the NBM, which may lead to changes in the free water within the NBM nucleus. Our findings suggest that impaired olfaction indicates early NBM degeneration, which could be detected by free water imaging.

Receiver operating characteristic curve analysis indicates that free water of the NBM can be applied to identify PD patients with hyposmia with a high sensitivity (81.6%). Although acetylcholinesterase PET directly measures cholinergic function, its application is limited by high cost, limited availability, and radiation exposure risks (Rispoli et al., 2018). In comparison, MRI can be utilized in large-scale population-based studies for screening purposes and has demonstrated considerable promise. Moreover, the resolution of MRI is much higher than that of PET, and it can display tiny anatomical structures, such as the NBM.

Our study indicated that patients with PD showed significantly decreased DAT binding in the caudate and putamen compared to healthy controls using DAT imaging. DAT is located in dopaminergic neurons and plays a key role in transporting free dopamine from the synaptic space back into the presynaptic terminal, helping to regulate dopamine concentrations (Kurosaki et al., 2003). In PD, the degeneration of dopaminergic neurons leads to a marked decrease in both DAT binding and dopamine levels (Uhl, 2003). The result of decreased DAT binding in PD in our study is consistent with this finding. Our study further showed that NBM free water was negatively correlated with DAT binding in the caudate and putamen, while NBM volume was positively correlated with DAT binding in the caudate and putamen. These results indicate that NBM degeneration may be correlated with reduced DAT binding. The pathophysiological changes in PD are notably complex, involving multiple neurotransmitter systems. Beyond the pronounced early impairment of the dopaminergic system, early damage to the cholinergic system has also been observed (Eisenstein et al., 2025). Our finding is in line with the previous pathological study showing that lewy body pathologies are associated with severe degeneration of the cholinergic neurons in the basal forebrain (Candy et al., 1983). This may reflect the synergistic degeneration of different neurotransmitter systems during the progression of neuro-pathological changes in PD. Moreover, previous studies suggest that presynaptic accumulation of α-synuclein can trigger synaptic dysfunction and ultimately lead to the loss of dendritic spines in postsynaptic neurons (Candy et al., 1983; Lippa et al., 1999). Therefore, the propagation of α-synuclein from the substantia nigra and basal ganglia—regions whose neurons directly project to cholinergic cells in the basal forebrain—may drive the degeneration of the basal forebrain cholinergic system (Candy et al., 1983; Lippa et al., 1999).

The main strength of this study is the large sample enrolled from an international multicenter cohort study. However, this study has some limitations. First, as this study was conducted retrospectively, the estimation of the bi-tensor model was based on single-shell diffusion data. When obtaining multi-shell diffusion data, it is possible to employ more advanced diffusion models to enhance the precision of tissue microstructure evaluation. Second, this research applied ComBat to harmonize the measures across various sites and scanners. However, potential nonlinearities and interactive effects between cohorts may require more advanced methods, such as nonlinear models, to identify and correct them effectively. Third, although we have limited the analyses within the segmented gray matter and excluded the voxels with high FA, the diffusion metrics may still be affected by the nearby white matter and cerebrospinal fluid. Fourth, due to the lack of longitudinal data to establish temporality, this study cannot answer this question whether free water change precedes olfactory decline.

## Conclusion

In conclusion, this study demonstrated that the free water of the NBM was associated with worse olfaction in idiopathic patients with PD. Our findings suggest that the free water of the NBM has the potential to provide early biomarkers of olfaction dysfunction in idiopathic patients with PD.

## Data Availability

The original contributions presented in this study are included in this article/supplementary material, further inquiries can be directed to the corresponding authors.

## Footnotes

1
https://www.ppmi-info.org/data

2
https://www.fil.ion.ucl.ac.uk/spm/software/spm12/

3
https://neuro-jena.github.io/cat/

4
https://www.mrtrix.org/

5
https://fsl.fmrib.ox.ac.uk/fsl/fslwiki

6
https://markvcid.partners.org/markvcid1-protocols-resources
